# Association between smoking and incident back pain: A prospective cohort study with 438 510 participants

**DOI:** 10.7189/jogh.13.04152

**Published:** 2023-11-22

**Authors:** Hao-Ran Xu, Yong-Hui Zhang, Thanh Luan Ngo, Qi-Hao Yang, Shu-Hao Du, Xue-Qiang Wang

**Affiliations:** 1 Department of Rehabilitation Medicine, The Second Affiliated Hospital of Wenzhou Medical University, Wenzhou, Zhejiang, China; 2 Department of Sport Rehabilitation, Shanghai University of Sport, Shanghai, China; 3 Department of Rehabilitation Medicine, Shanghai University of Medicine and Health Sciences Affiliated Zhoupu Hospital, Shanghai, China

## Abstract

**Background:**

Although smoking is a known potential contributor to back pain, there is still a lack of quantitative studies on the effects of different doses on back pain (BP) occurrence, including a lack of a longitudinal cohorts. To address this gap, we aimed to investigate the association between various smoking-related exposures and back pain incidence to advance global efforts toward smoking cessation and guide primary prevention of BP.

**Methods:**

In this prospective cohort study, we retrieved data on 438 510 patients from the UK Biobank who were free of back pain and who were recruited in 2006-2010, and followed them up from baseline through 1 April 2022. We extracted data on smoking-related exposures, including smoking status (SS), number of cigarettes smoked daily (CPD), and pack-years of own smoking (PY) and examined back pain incidence as an outcome. We used a Cox proportional hazard model adjusted for several covariates, multiple imputation methods, and population attribution fraction.

**Results:**

During the median follow-up of 12.98 years, 31 467 participants developed BP, with incidence rates in former and current smokers of 1.13 (95% confidence interval (CI) = 1.10-1.16, *P* < 0.000) and 1.50 (95% CI = 1.45-1.56, *P* < 0.000), respectively. The hazard ratios (HRs) of participants who smoked more than 30 CPD and those with more than 30 PY were 1.45 (95% CI = 1.36-1.55, *P* < 0.000) and 1.45 (95% CI = 1.40-1.50, *P* < 0.000), respectively. Relative to male, female smokers were at more risk of developing BP. Not smoking, quitting smoking, and reducing CPD and PY could lower the BP risk by 7.8%, 5.4%, 9.8%, and 18.0%, respectively.

**Conclusions:**

Ever smoking, higher cigarette consumption daily, and increased smoking intensity were associated with an increased BP risk. This association was stronger in female smokers. Not smoking, smoking cessation, and reducing smoking volume and intensity were effective measures to prevent BP occurrence.

Back pain (BP) is a prevalent global health issue affecting millions of individuals of all age groups worldwide [1]. Categorised as “dorsalgia” in the International Statistical Classification of Diseases and Related Health Problems 10^th^ Revision (ICD-10), it refers to pain or discomfort in the dorsal (back) region of the body, which may stem from abnormalities in spinal vertebrae, back muscles, tendons, ligaments, or neural structures. In 2015, BP was associated with over 60 million disability-adjusted life years, representing a 54% increase since 1990, with the most substantial rise occurring in low- and middle-income countries [2]. It is also the leading cause of productivity loss in both developed and developing nations [3]. While its etiology is not yet fully understood, determining its risk factors is key to prevention, especially due to difficulties with fully treating it and its high rate of occurrence [4].

Smoking remains a significant public health concern worldwide, with around 47.1 million adult smokers in the USA alone in 2020 [5,6]. It is a potential contributor to various adverse health consequences, including BP [6], and is closely related to various types of pain [7,8]. One study found that current smokers had an increased incidence of pain in various body regions compared with never smokers, with the highest incidence observed in the occurrence of BP [9]. Several studies have investigated the association between smoking-related factors and BP incidence, with consistent findings that smokers have a higher risk compared with never smokers [10-12]. However, most of these studies used a cross-sectional design, which may not capture the temporal effects of exposures on outcome, or had small sample sizes, while others were outdated and thus possibly had suspect reliability of results. Additionally, existing research has predominantly focused on investigating the impact of smoking status (current vs never) on BP incidence, without quantitatively exploring the effects of different smoking doses on BP occurrence.

In this prospective cohort study, we aim to investigate the association between various smoking-related exposures, such as smoking status (SS), number of cigarettes smoked daily (CPD), pack-years of own smoking (PY), and BP incidence based on the UK Biobank database, which comprises a longitudinal population-based cohort. By analyzing data from over 430 000 participants, we sought to improve the understanding of the temporal relationship between the incidence of BP and various smoking-related exposures over time and address the aforementioned evidence gap. Understanding the influence of smoking doses on BP incidence could facilitate personalised interventions for individuals with varying doses of smoking, aimed at preventing BP, and could offer practical implications for promoting global smoking cessation and guiding primary prevention of BP in clinical settings.

## METHODS

### Study design and population

In this population-based cohort study, we extracted baseline data from the UK Biobank, comprising over 500 000 individuals aged 37-73 whose information was collected in 2006-2010. We excluded those diagnosed with BP before baseline (n = 47 299), without exposure data (n = 16 224), or with abnormal smoking data (n = 334), resulting in a sample of 438 510 individuals (Figure S1 in the **Online Supplementary Document**). All data used in the study are publicly available. We obtained ethics approval from the North West Multi-Center Research Ethics Committee (ref: 11/NW/0382), and all participants signed informed consent forms. Patients or the public were not involved in the design, conduct, or reporting, or dissemination plans of our research.

### Exposure

We obtained all data on smoking-related exposures (SS (Field ID: 20116), CPD (Field ID: 3456, 2887, and 6183), and PY (Field ID: 20161) from a touchscreen questionnaire in the UK Biobank Assessment Centres. SS was a categorical variable comprising never smokers (never smoked or smoked <100 times in their lifetime), former smokers (smoked in the past or those smoked >100 times in their lifetime), and current smokers (smoking at the time of data collection) [13-15]. To avoid potential bias due to different tobacco types, we excluded participants who previously smoked cigars (or cigarettes) but currently smoked cigarettes (or cigars). CPD was the average number of cigarettes smoked daily. We excluded participants with CPD>60 due to potential data abnormality [13]. PY is a volumetric composite reflecting smoking intensity and containing both temporal (years) and quantitative (pack) information, calculated as the number of cigarettes smoked daily, divided by twenty, multiplied by the years of smoking [16]. Both CPD and PY can be treated as continuous and categorical variables (“None”, “Up to 10”, “Between 11 and 20”, “Between 21 and 30”, and “More than 30”) according to different analyses [13].

### Outcome

Our primary outcome of interest was BP incidence, defined as dorsalgia (M54 in the International Classification of Diseases, 10th revision (ICD-10)), involving panniculitis affecting regions of neck and back, radiculopathy, cervicalgia, sciatica, lumbago with sciatica, low BP, pain in thoracic spine, other dorsalgia, and unspecified dorsalgia (Table S1 in the **Online Supplementary Document**). The date of the first occurrence of primary outcome was collected from primary care, hospital admissions, and self-report (ID 131928). The follow-up started from the date of baseline assessment and ended at the date of first BP incidence, death, or termination of follow-up (1 April 2022) based on the status of every participant.

### Covariates

Our covariates were age, sex (male and female), ethnicity (White, Mixed, Chinese, South Asian, Black, and other), body mass index (BMI) categories (underweight, normal, overweight, and obese), educational level (college, professional qualifications, and other), Townsend deprivation index (continuous variable), and sedentary behavior (SB) (continuous variable, calculated by summing the time spent driving, using a computer, and watching television in a typical day).

### Statistical analyses

According to different SS, we presented the participants’ demographic and baseline information as means with standard deviations (SDs) for continuous variables and numbers with percentages for categorical variables. We applied multiple imputation with chained equations to missing covariates through a predictive mean matching built in R, version 4.2.2 (R Core Team, Vienna, Austria).

We examined the hazard ratio (HR) and its 95% confidence interval (CI) between smoking-related exposure (SS, CPD, and PY as categorical variables) and BP incidence using Cox proportional hazards regression models, after which we analyzed CPD and PY as continuous variables to detect the dose-response relationship between smoking dose and BP incidence by using restricted cubic splines fitted in Cox proportional hazard regression models. We estimated the cumulative hazard rate between smoking-related exposures (SS, CPD, and PY as categorical variables) and incident BP by follow-up by applying the Nelson Aalen estimator, a non-parametric statistical method which helps in the visualization and analysis of the cumulative hazard curve in survival analysis. We adjusted all analyses by the covariates listed above and stratified the Cox analyses by sex (male and female), age (≤60 and >60), and BMI (<30 and ≥30) to further examine the association between smoking and BP incidence.

We used population-attributable fraction (PAF) analysis to estimate the proportion of risks which could be prevented by not smoking (never vs current and former), quitting smoking (former vs current), and reducing smoking volume and intensity (“Up to 10” vs more smoking volume and intensity).

We also performed several sensitivity analyses to ensure the robustness of primary results by repeating the Cox proportional hazards regression models after excluding participants with missing covariates to avoid any bias from imputed covariates, and separately after excluding participants with self-reported chronic BP at baseline assessment because those with pain initially may be more likely to be diagnosed as dorsalgia during the follow-up, regardless of their smoking behaviors.

We conducted all analyze in R, version 4.2.2 (R Core Team, Vienna, Austria). We considered *P*-values <0.05 as statistically significant.

## RESULTS

The participants had a mean age of 56.4 (SD = 8.12) years and were mainly women (54.9%). BP occurred in 31 467 (7.2%) cases during follow-up (median = 12.98 years, interquartile range (IQR) = 12.10-13.72). Never smokers were more likely to have higher economic status and educational level., while former and current smokers were more sedentary in daily life. Former smokers had a mean CPD of 18.8 (SD = 9.85) and a mean PY of 20.7 (SD = 17.0), while current smokers had higher values for both variables (CPD = 20.7 (SD = 17.0)), PY = 28.0 (SD = 17.3)) (**Table 1**).

**Table 1:** Baseline characteristics of participants stratified by smoking status*

	Never (n = 248 128)	Former (n = 158 123)	Current (n = 32 259)	Overall (n = 438 510)
**Age (years), mean (SD)**	55.8 (8.17)	57.7 (7.84)	54.5 (8.07)	56.4 (8.12)
**Sex**				
Female	148 343 (59.8)	76 074 (48.1)	16 229 (50.3)	240 646 (54.9)
Male	99 785 (40.2)	82 049 (51.9)	16 030 (49.7)	197 864 (45.1)
**Back pain occurrence**				
Yes	16 166 (6.5)	12 120 (7.7)	3181 (9.9)	31 467 (7.2)
No	231 962 (93.5)	146 003 (92.3)	29 078 (90.1)	407 043 (92.8)
**Ethnicity**				
White	231 281 (93.2)	153 044 (96.8)	30 407 (94.3)	414 732 (94.6)
Mixed	1316 (0.5)	926 (0.6)	376 (1.2)	2618 (0.6)
Chinese	1162 (0.5)	203 (0.1)	79 (0.2)	1444 (0.3)
South Asian	6613 (2.7)	1422 (0.9)	490 (1.5)	8525 (1.9)
Black	3391 (1.4)	1052 (0.7)	467 (1.4)	4910 (1.1)
Other	4365 (1.8)	1476 (0.9)	440 (1.4)	6281 (1.4)
**BMI categories**				
Underweight	1333 (0.5)	472 (0.3)	455 (1.4)	2260 (0.5)
Normal	87 550 (35.3)	43 871 (27.7)	12 042 (37.3)	143 463 (32.7)
Overweight	103 259 (41.6)	70 882 (44.8)	12 854 (39.8)	186 995 (42.6)
Obese	55 986 (22.6)	42 898 (27.1)	6908 (21.4)	105 792 (24.1)
**Educational level**				
College	91 042 (36.7)	48 388 (30.6)	6148 (19.1)	145 578 (33.2)
Professional qualifications	37 104 (15.0)	22 792 (14.4)	3869 (12.0)	63 765 (14.5)
Others	119 982 (48.4)	86943 (55.0)	22 242 (68.9)	229 167 (52.3)
**Townsend deprivation index, mean (SD)**	-1.60 (2.94)	-1.23 (3.08)	0.452 (3.55)	-1.31 (3.09)
**Sedentary behavior, mean (SD)**	4.62 (2.35)	5.02 (2.44)	5.45 (2.95)	4.82 (2.44)
**Number of cigarettes smoked daily, mean (SD)**	NA	18.8 (9.85)	15.4 (8.26)	NA
**Pack-years of own smoking, years, mean (SD)**	NA	20.7 (17.0)	28.0 (17.3)	NA

SD – standard deviation, BMI – body mass index, NA – not applicable

*Data are presented as n (%), unless specified otherwise.

Smoking-related exposures (SS, CPD, and PY) were closely associated with BP risk (**Table 2**). Former (1.13%; 95% CI = 1.10-1.16) and current smokers (1.50; 95% CI = 1.45-1.56) had a higher incidence of BP compared to never smokers.

**Table 2:** The association between different smoking-related exposures and incident back pain*

Categories	n/N events	HR (95% CI)
**Smoking status**		
Never	248 128/16 166	1 (ref)
Former	158 123/12 120	1.13 (1.10-1.16)
Current	32 259/3181	1.50 (1.45-1.56)
*P*-value for trend	*P *< 0.000	1.19 (1.17-1.21)
**Number of cigarettes smoked daily**		
None	303 681/19 936	1 (ref)
Up to 10	38 702/2916	1.12 (1.08-1.17)
Between 11 and 20	70 267/6042	1.24 (1.20-1.28)
Between 21 and 30	17 187/1650	1.37 (1.30-1.44)
More than 30	8673/923	1.45 (1.36-1.55)
*P*-value for trend	*P *< 0.000	1.11 (1.10-1.12)
HR per 5 units	*P *< 0.000	1.05 (1.05-1.06)
HR per 10 units	*P *< 0.000	1.10 (1.11-1.12)
**Pack-years of own smoking **		
None	306 012/20 156	1 (ref)
Up to 10	36 398/2411	1.03 (0.98-1.07)
Between 11 and 20	35 975/2867	1.17 (1.13-1.22)
Between 21 and 30	25 697/2387	1.32 (1.27-1.38)
More than 30	34 428/3646	1.45 (1.40-1.50)
*P*-value for trend	*P *< 0.000	1.10 (1.09-1.10)
HR per 5 units	*P *< 0.000	1.04 (1.04-1.04)
HR per 10 units	*P *< 0.000	1.08 (1.07-1.09)

*All hazard ratios (HRs) and their 95% confidence intervals (CIs) were derived from a Cox proportional hazards regression model adjusted for age, sex, ethnicity, body mass index, educational level, Townsend Deprivation Index, and sedentary behavior.

Relative to participants who did not smoke daily, those who smoked up to 10 CPD had a HR of 1.12 (95% CI = 1.08-1.17), while those who smoked more than 30 CPD had a HR of 1.45 (95% CI = 1.36-1.55). With each increase in CPD by one category, the incidence of BP increased by 11% (*P* for trend <0.001), while smoking an additional half-pack of cigarettes (10-unit increase) per day increased BP risk by 10% (95% CI = 1.11-1.12). Compared with participants who had no PY, the HR among participants with PY between 11 and 20 was 1.17 (95% CI = 1.13-1.22) and more than 30 PY was 1.45 (95% CI = 1.40-1.50). With each increase in PY by one category, the incidence of BP increased by 10% (*P* for trend <0.001), with every 10-unit increase leading to an 8% (95% CI = 1.07-1.09) rise in BP risk.

**Figure 1:**
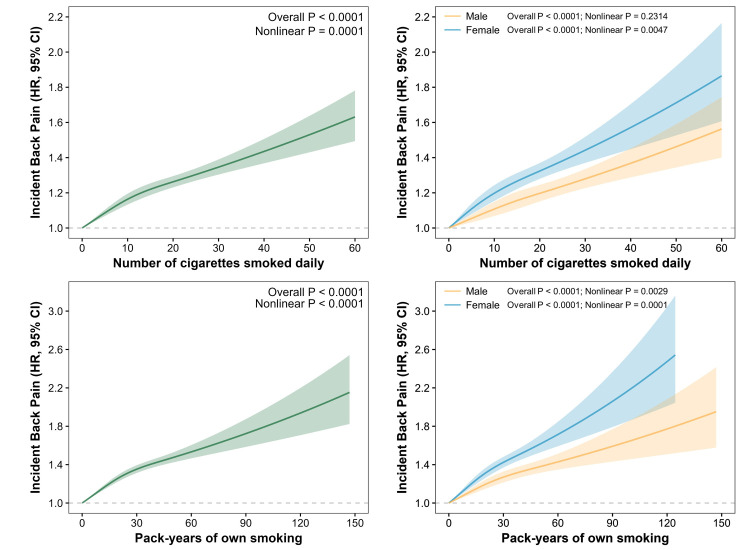
Dose-response associations between smoking dose (number of cigarettes smoked daily and pack-years of own smoking) and incident back pain in all, male, and female participants. Data presented as hazard ratios and 95% confidence intervals. We adjusted models investigating the association between smoking dose and BP risk in all participants for age, sex, ethnicity, BMI, educational level, Townsend Deprivation Index, and sedentary behavior, and those investigating the association between smoking dose and BP risk in male and female participants for age, ethnicity, BMI, educational level, Townsend Deprivation Index, and sedentary behavior. The dose-response association between smoking dose and incident back pain was significant (*P* < 0.0001) and nonlinear (nonlinear *P* < 0.0001).

The dose-response association between CPD, PY, and BP incidence in all, male, and female participants were significant (*P* < 0.001 for all cases) and nonlinear (*P* < 0.001) (**Figure 1**). Overall, higher CPD and PY were related to an increase in BP incidence, but the association was nonlinear, as evidenced by the change in the slope of increase before and after change points. This means that the risk of BP incidence increased more rapidly before the change points around 10 CPD and around 30 PY, respectively. Women had a higher risk of developing BP relative to men, with the same dose of smoking.

**Figure 2:**
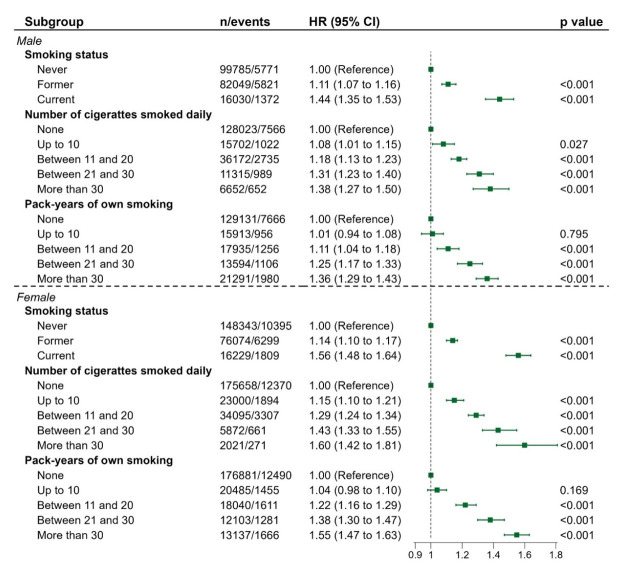
Association between different smoking-related exposures and incident BP stratified by sex. We derived all hazard ratios and their 95% confidence intervals from Cox proportional hazards regression, after adjusting for age, ethnicity, BMI, educational level, Townsend Deprivation Index, and sedentary behavior.

Overall, more smoking was related to increasing BP incidence, regardless of sex. Male current smokers had a 44% (95% CI = 1.35-1.53) higher risk of developing BP than male never smokers, while the HRs for males who smoked more than 30 CPD and with more than 30 PY were 1.38 (95% CI = 1.27-1.50) and 1.36 (95% CI = 1.29-1.43), respectively. Meanwhile, female current smokers had a 56% (95% CI = 1.48-1.64) more risk compared with female never smokers, while the HRs of females who smoked more than 30 CPD and with more than 30 PY were 1.60 (95% CI = 1.42-1.81) and 1.55 (95% CI = 1.47-1.63), respectively. Under the same circumstance of smoking more than 30 CPD, female smokers had an additional 22% susceptibility to develop BP compared with male smokers, and an additional 19% risk among female smokers with more than 30 PY, relative to male smokers under equal condition. The association between smoking and BP incidence stratified by age and BMI showed no obvious risk difference between subgroups (**Figure 2**, Table S2 and Table S3 in the **Online Supplementary Document**).

**Figure 3:**
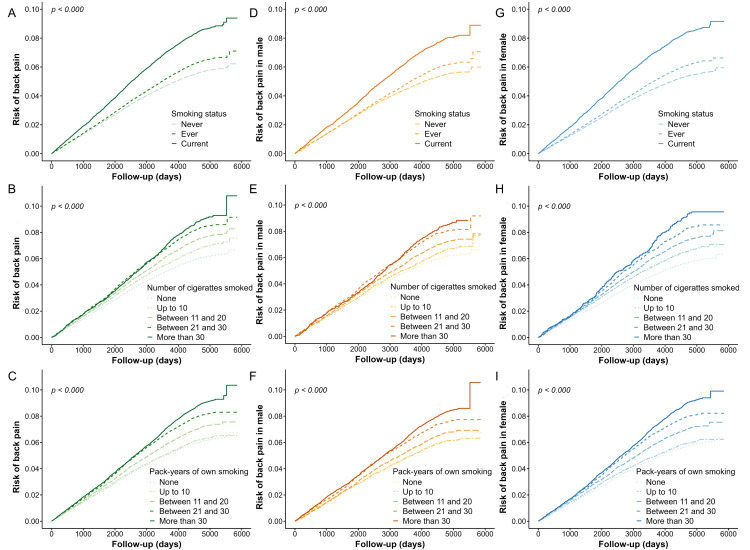
Cumulative risk of BP incidence for smoking-related exposures in all, male and female participants. We adjusted models investigating the cumulative risk of BP incidence for smoking-related exposures in all participants for age, sex, ethnicity, BMI, educational level, Townsend Deprivation Index, and sedentary behavior, and those investigating the cumulative risk of BP incidence for smoking-related exposures in male and female participants for age, ethnicity, BMI, educational level, Townsend Deprivation Index, and sedentary behavior. Panels A-C show the cumulative back pain risk in different smoking-related exposures categories (smoking status, cigarettes per and pack-years) during a 6000-day (16.44 years) follow-up among all participants. Panels D-F and G-I show the outcomes of further subgroup analysis among male and female participants, respectively.

Current smokers had a 3000-day (i.e. 8.22 years) and a 6000-day (i.e. 16.44 years) cumulative BP incidence of 4%-6% and 8%-10%, respectively. Compared with participants who did not smoke daily, those who smoked more than 30 CPD had an approximately 4%-5% higher 6000-day cumulative BP risk. Similarly, participants with more than 30 PY had 4%-5% higher 6000-day cumulative BP risk than those without PY. The cumulative risk of sex subgroups between smoking-related exposure with BP incidence indicated a consistent trend with that among all participants (**Figure 3**, Panels A-I).

Our estimates showed that not smoking (7.8%; 95% CI = 6.8-8.8) and quitting smoking (5.4%; 95% CI = 4.5-6.2) could lower the risk of BP; while it could reducing smoking volume (9.8%; 95% CI = 6.9-12.7) and intensity (18.0%; 95% CI = 15.0-21.0) from more than 30 to down to 10 (**Table 3**). The results of two sensitivity analyses demonstrated no substantial bias after excluding participants with missing and participants with chronic BP at baseline (Tables S4-S5 in the **Online Supplementary Document**).

**Table 3:** Population attributable fraction

PAF	Back pain incidence (95% CI)	*P*-value
Attributable to current and former smoking behaviors (vs never smoke)	7.8% (6.8-8.8)	<0.001
Attributable to current smoking behaviors (vs former smoke)	5.4% (4.5-6.2)	<0.001
Attributable to more cigarettes smoked daily (vs number of cigarettes smoked daily was up to 10)	9.8% (6.9-12.7)	<0.001
Attributable to more Pack-years of own smoking (vs pack-years of own smoking was up to 10)	18.0% (15.0-21.0)	<0.001

PAF – population-attributable fraction, CI – confidence interval

*Population-attributable fraction (PAF) analysis was used to estimate the proportion of risks which could be prevented by not smoking (never vs current and former), quit smoking (former vs current) and reducing smoking volume and intensity (‘’Up to 10” vs more smoking volume and intensity).

## DISCUSSION

Through this prospective cohort study with over 430 000 participants, we aimed to verify the associations between smoking and BP incidence. Our findings confirm that ever smoking, a greater number of cigarettes smoked daily and higher smoking intensity are related to the developing of BP, independent of various confounding factors. In particular, current smokers, smoked over 30 cigarettes daily and PY over 30 and were strongly associated with 50%, 45%, and 45% more risk of BP, respectively. This association was stronger in female smokers relative to male smokers, without significant risk differences by age and BMI. More smoking-related behaviors are associated with a 4%-10% higher 6000-day cumulative risk of developing BP. Meanwhile, we detected a nonlinear, but overall increasing dose-response relationship between smoking dose and BP risk. Not smoking, quitting smoking, and lowering smoking volume and intensity could alleviate BP risk by 7.8%, 5.4%, 9.8% and 18.0%, respectively.

Our primary findings revealed former smokers had a higher risk of BP than never smokers, but a much lower one than current smokers, generally consistent with existing literature, such as a meta-analysis which pooled the odds ratio (OR) from several cross-sectional studies that investigated smoking and incident BP [17]. Based on their pooled findings, current and former smokers were more likely to develop BP, with current smokers more susceptible to BP compared with former smokers (pooled OR = 1.33; 95% CI = 1.26-1.41 vs OR = 1.20; 95% CI = 1.00-1.44). Another study from a British national survey with 12 907 subjects had similar findings, but observed a lack of a significant risk difference between current and former smokers [18]. This difference could result from the different designs and sample sizes between studies. We thus used a longitudinal rather than a cross-sectional design to better capture the temporal effects of smoking on BP incidence, while also obtaining a relatively larger sample size to improve the accuracy of our results. Our findings may indirectly implicate the significance of smoking cessation in preventing the occurrence of BP.

To date, relatively few studies have quantified the association between the dose of cigarette smoking and incident BP. We addressed this gap by grouping smoking dose into CPD and PY and found an overall increasing dose-response relationship between the two variables, with more smoking dose being related to an increase in BP risk. Moreover, people who smoked an additional half pack of cigarettes per day would have their BP risk increased by 10%. A northern Finland Birth Cohort study on 1987 adolescents reported an extremely high BP risk among female adolescents who smoked over nine cigarettes daily (OR = 2.80; 95% CI = 1.11-7.09) and whose PY was over 1.5 (OR = 2.20; 95% CI = 1.13-4.28), while the associations were insignificant for male adolescents [10]. This agrees with our findings that increased smoking dose is related to more BP risk and that this association is stronger among female smokers, even in consistent smoking conditions. Different studies have described the role of gender in tobacco pathobiology, with females being more vulnerable to adverse health events than males [19,20]. Altered levels of female hormone (estrogen) may explain this finding. Smoking could mediate toxic effects and decrease estrogen secretion, which has been proven to be a contributor to low bone quality and mass, thereby leading to BP [21-23]. Together with the existing evidence which emphasizes the higher prevalence of BP in females than males, female smokers should be given extra attention during primary prevention screening for BP and personalised patient education regarding lifestyle in clinical settings [3].

Thus far, the mechanisms underlying the role of smoking in the development of BP have been only partially determined. First, smoking is strongly associated with osteoporosis, which could induce vertebral bone density loss and bone microfractures. This long-term effect on bone tissues causes chronic inflammation and elevates the risk and extent of further spinal damage [24]. Meanwhile, BP is closely associated with the degeneration of the intervertebral disc (IVD) [25]. Smoking can impair the vascular circulation beneath the IVD, causing an inadequate supply of nutrients and growth factors to the disc tissue and making the disc becomes more vulnerable to mechanical force and deformation [26]. Furthermore, populations with smoking behaviors demonstrated a significantly lower pain threshold compared with that of non-smokers, indicating the over-amplification effect of smoking on pain perception signals [18,27]. Lastly, smoking behavior is related to mental disorders, such as anxiety and depression. It is hypothesized that nicotine may briefly alleviate symptoms of anxiety and depression. However, this relief is often short-lived, potentially leading to a worsening of mood [28]. Notably, anxiety and depression can exacerbate pain, while the presence of chronic pain can also contribute to the development or exacerbation of these emotional states [29]. This bidirectional relationship between smoking-induced psychological issues and pain is associated with core muscle dysfunction, pain catastrophizing, and inflammatory response, ultimately contributing to spinal-related pain [30]. Other potential factors, such as low physical activity, high BMI, sleep disorder, and overstress may interact with smoking, resulting in further BP [28,31-33].

Given the multi-perspective negative impacts of smoking on spinal health, we found that changes in smoking behavior, such as quitting smoking and not smoking, led to a decrease in BP risk, which is consistent with previous research [14,34], while lowering smoking volume and intensity resulted in a more significant BP risk reduction among current smokers. One potential explanation for this observation is the recovery process that occurs after prolonged nicotine consumption, in which the central nervous system undergoes a restorative phase, leading to a reduction in over-amplified pain perception following the discontinuation of nicotine exposure [35]. Thus, both smoking cessation and dose reduction might be simultaneously beneficial to lower the risk of BP in the future and the negative effects caused by smoking are partially reversible.

This study has several strengths, the first being its relatively large sample size which enhances increases the accuracy of our findings. Second, we adopted a prospective cohort design, thus partially complementing previous, predominantly cross-sectional studies. Furthermore, this study is the first to quantify the dose-response relationship between smoking doses and the occurrence of BP based on a substantial sample, addressing the current gap in evidence.

However, our study also has several limitations. Its longitudinal cohort nature prevents any causal inference; although we discovered an association between smoking and BP events, further high-quality randomized controlled trials, long-term follow-up studies, and evidence syntheses are needed to confirm causality. Despite the sensitivity analysis in which we excluded participants with self-reported chronic BP, reverse causation remains possible. Although we have excluded some participants with abnormal exposure data, the smoking-related information may not be fully accurate due to participants’ recall bias and mistakes made while filling in the touchscreen questionnaire. Additionally, most participants included were Caucasian, with a small minority of other races, so our findings cannot necessarily be generalized to other populations. Moreover, although many potential covariates have been adjusted, there could have been unknown confounding effects. Future research should use more appropriate designs to examine a possible causal association between smoking on BP among different racial and ethnic populations.

## CONCLUSIONS

We found ever smoking behavior, a greater number of cigarettes smoked daily and higher smoking intensity are related to an increased BP risk, while female smokers were more likely to develop BP compared with male smokers. Not smoking, quitting smoking, and lowering smoking volume and intensity are effective measures to prevent potential BP occurrence.

## Additional material


Online Supplementary Document
