# Supplementary material for: Association between smoking and incident back pain: A prospective cohort study with 438 510 participants
Source: J Glob Health. 2023 Nov 22;13:04152. doi: 10.7189/jogh.13.04152 (PMC10663706; doi:10.7189/jogh.13.04152)
Supplement: Online Supplementary Document [file jogh-13-04152-s001.pdf]

Figure S1 Flow chart

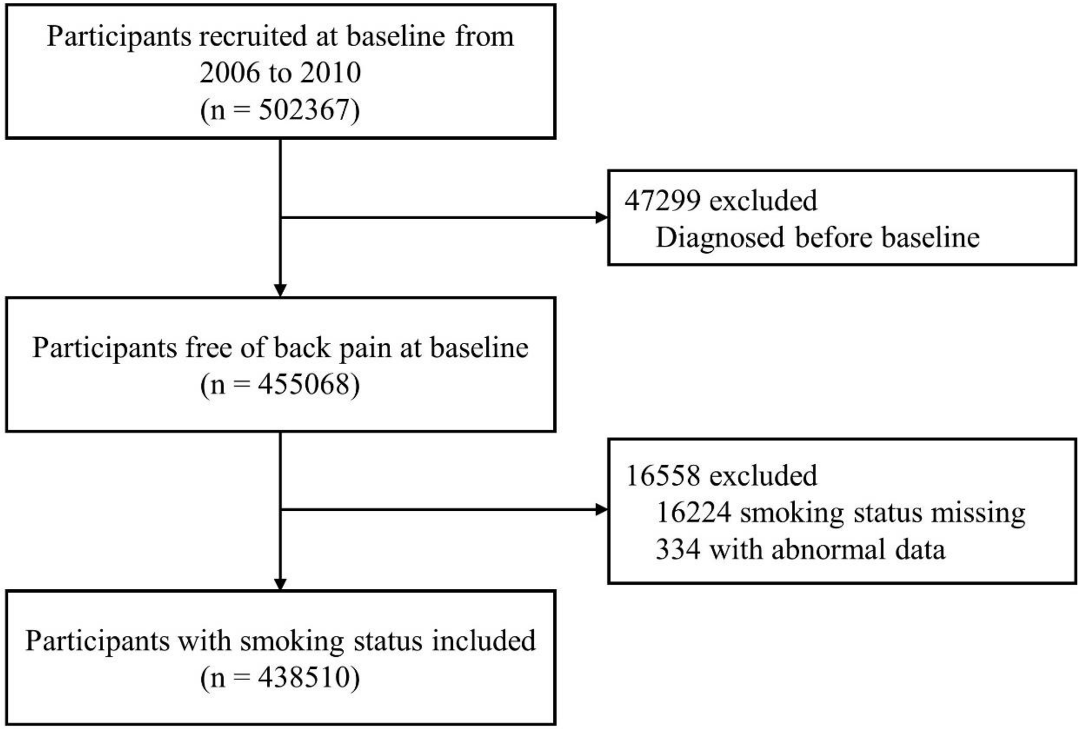

The data of 502367 participants aged 37-73 participants from 2006 to 2010 during baseline assessment were available in the UK Biobank. Participants diagnosed with BP before baseline (n=47,299), without SS data (n=16,224), or with abnormal smoking data (n=334) were excluded, which left 438,510 participants to further analyses.

Table S1 Disease definitions

ICD-10 Code	Classification	Brief Definition
M54	Dorsalgia (back pain)	General code for various forms of back pain and related conditions.
M54.1	Radiculopathy	Pain due to compression or irritation of a spinal nerve, often radiating.
M54.2	Cervicalgia	Neck pain, often associated with the cervical spine.
M54.3	Sciatica	Pain that radiates along the sciatic nerve, typically from the lower back.
M54.4	Lumbago with sciatica	Lower back pain with radiating pain along the sciatic nerve.
M54.5	Low back pain	Pain localized in the lower back region.
M54.6	Pain in thoracic spine	Pain occurring in the mid-back area around the thoracic spine.
M54.8	Other dorsalgia	Code for other specified forms of back pain conditions.
M54.9	Dorsalgia, unspecified	Code for unspecified or undifferentiated back pain.

The Code is following ICD-10 by World Health Organization (WHO). The related information was available in https://biobank.ndph.ox.ac.uk/showcase/field.cgi?id=41270

Table S2 The association between different smoking-related exposures and incident back pain stratified by age

Smoking Status			Number of cigarettes smoked daily			Pack-Years of own Smoking		
Categories	n/events	HR (95%CI)	Categories	n/events	HR (95%CI)	Categories	n/events	HR (95%CI)
**Aged <= 60 (n=273006)**								
Never	162279/9564	1 (ref)	None	195605/11560	1 (ref)	None	196889/11664	1 (ref)
Former	87536/5862	**1.11 (1.08-1.15)**	Up to 10	23244/1578	**1.12 (1.06-1.18)**	Up to 10	23698/1420	1.02 (0.96-1.07)
Current	23191/2222	**1.52 (1.45-1.59)**	Between 11 and 20	40727/3255	**1.28 (1.23-1.33)**	Between 11 and 20	21831/1675	**1.24 (1.18-1.30)**
P for trend	***p* < 0.000**	**1.20 (1.17-1.22)**	Between 21 and 30	9530/861	**1.41 (1.31-1.51)**	Between 21 and 30	14956/1336	**1.39 (1.32-1.48)**
			More than 30	3900/394	**1.51 (1.36-1.67)**	More than 30	15632/1553	**1.53 (1.45-1.61)**
			P for trend	***p* < 0.000**	**1.12 (1.11-1.14)**	P for trend	***p* < 0.000**	**1.11 (1.10-1.13)**
			HR per 5 units	***p* < 0.000**	**1.06 (1.05-1.07)**	HR per 5 units	***p* < 0.000**	**1.05 (1.05-1.06)**
**Aged > 60 (n=165504)**								
Never	85849/6602	1 (ref)	None	108076/8376	1 (ref)	None	109123/8492	1 (ref)
Former	70587/6258	**1.17 (1.13-1.21)**	Up to 10	15458/1338	**1.12 (1.06-1.19)**	Up to 10	12700/991	1.04 (0.97-1.11)
Current	9068/959	**1.43 (1.33-1.53)**	Between 11 and 20	29540/2787	**1.22 (1.17-1.28)**	Between 11 and 20	14144/1192	**1.09 (1.03-1.16)**
P for trend	***p* < 0.000**	**1.18 (1.15-1.22)**	Between 21 and 30	7657/789	**1.37 (1.27-1.47)**	Between 21 and 30	10741/1051	**1.25 (1.17-1.33)**
			More than 30	4773/529	**1.47 (1.34-1.60)**	More than 30	18796/2093	**1.46 (1.38-1.53)**
			P for trend	***p* < 0.000**	**1.10 (1.09-1.12)**	P for trend	***p* < 0.000**	**1.09 (1.08-1.10)**
			HR per 5 units	***p* < 0.000**	**1.05 (1.04-1.06)**	HR per 5 units	***p* < 0.000**	**1.04 (1.03-1.04)**
			HR per 10 units	***p* < 0.000**	**1.10 (1.08-1.12)**	HR per 10 units	***p* < 0.000**	**1.08 (1.07-1.09)**

Participants were divided into two subgroups based on age (<= 60 and > 60). All hazard ratios (HRs) and its 95% confident intervals (CIs) were derived from Cox proportional hazards regression. The analyses were adjusted for sex, ethnicity, BMI, educational level, Townsend Deprivation Index and sedentary behaviour.

Table S3 The association between different smoking-related exposures and incident back pain stratified by BMI

Smoking Status			Number of cigarettes smoked daily			Pack-Years of own Smoking		
Categories	n/events	HR (95%CI)	Categories	n/events	HR (95%CI)	Categories	n/events	HR (95%CI)
**BMI<30 (n=332718)**								
Never	192142/11297	1 (ref)	None	235142/13962	1 (ref)	None	236814/14117	1 (ref)
Former	115225/7921	**1.14 (1.10-1.17)**	Up to 10	31024/2188	**1.14 (1.09-1.20)**	Up to 10	29010/1759	1.02 (0.97-1.07)
Current	25351/2349	**1.53 (1.46-1.60)**	Between 11 and 20	50827/4010	**1.27 (1.22-1.32)**	Between 11 and 20	26557/1976	**1.22 (1.16-1.28)**
P for trend	***p* < 0.000**	**1.20 (1.17-1.22)**	Between 21 and 30	11036/981	**1.44 (1.35-1.54)**	Between 21 and 30	17976/1564	**1.39 (1.32-1.46)**
			More than 30	4689/426	**1.45 (1.32-1.60)**	More than 30	22361/2151	**1.49 (1.42-1.56)**
			P for trend	***p* < 0.000**	**1.12 (1.11-1.14)**	P for trend	***p* < 0.000**	**1.11 (1.09-1.12)**
			HR per 5 units	***p* < 0.000**	**1.06 (1.05-1.07)**	HR per 5 units	***p* < 0.000**	**1.05 (1.04-1.05)**
			HR per 10 units	***p* < 0.000**	**1.12 (1.11-1.14)**	HR per 10 units	***p* < 0.000**	**1.09 (1.08-1.10)**
**BMI>=30 (n=105792)**								
Never	55986/4869	1 (ref)	None	68539/5974	1 (ref)	None	69198/6039	1 (ref)
Former	42898/4199	**1.13 (1.08-1.18)**	Up to 10	7678/728	1.07 (0.99-1.15)	Up to 10	7388/652	1.05 (0.96-1.13)
Current	6908/832	**1.40 (1.30-1.51)**	Between 11 and 20	19440/2032	**1.20 (1.14-1.26)**	Between 11 and 20	9418/891	**1.10 (1.03-1.18)**
P for trend	***p* < 0.000**	**1.16 (1.12-1.20)**	Between 21 and 30	6151/669	**1.28 (1.18-1.39)**	Between 21 and 30	7721/823	**1.22 (1.14-1.32)**
			More than 30	3984/497	**1.46 (1.33-1.60)**	More than 30	12067/1495	**1.40 (1.32-1.49)**
			P for trend	***p* < 0.000**	**1.09 (1.08-1.11)**	P for trend	***p* < 0.000**	**1.08 (1.07-1.10)**
			HR per 5 units	***p* < 0.000**	**1.04 (1.04-1.05)**	HR per 5 units	***p* < 0.000**	**1.03 (1.03-1.04)**
			HR per 10 units	***p* < 0.000**	**1.09 (1.07-1.11)**	HR per 10 units	***p* < 0.000**	**1.07 (1.05-1.08)**

Participants were divided into two subgroups based on BMI (< 30 and >=30). All hazard ratios (HRs) and its 95% confident intervals (CIs) were derived from Cox proportional hazards regression. The analyses were adjusted for age, sex, ethnicity, educational level, Townsend Deprivation Index and sedentary behaviour.

Table S4 The association between different smoking-related exposures and incident back pain after excluding missing covariates

Smoking Status			Number of cigarettes smoked daily			Pack-Years of own Smoking		
Categories	n/events	HR (95%CI)	Categories	n/events	HR (95%CI)	Categories	n/events	HR (95%CI)
Never	235637/15006	1 (ref)	None	289025/18577	1 (ref)	None	290209/18688	1 (ref)
Former	151285/11446	**1.13 (1.11-1.16)**	Up to 10	36688/2709	**1.12 (1.08-1.17)**	Up to 10	35147/2298	1.03 (0.99-1.08)
Current	29881/2908	**1.51 (1.45-1.58)**	Between 11 and 20	66699/5656	**1.25 (1.21-1.28)**	Between 11 and 20	34476/2706	**1.18 (1.13-1.22)**
P for trend	***p* < 0.000**	**1.19 (1.17-1.21)**	Between 21 and 30	16245/1552	**1.38 (1.31-1.46)**	Between 21 and 30	24449/2240	**1.33 (1.27-1.39)**
			More than 30	8146/866	**1.47 (1.37-1.58)**	More than 30	32522/3428	**1.47 (1.41-1.53)**
			P for trend	***p* < 0.000**	**1.11 (1.10-1.12)**	P for trend	***p* < 0.000**	**1.10 (1.09-1.11)**
			HR per 5 units	***p* < 0.000**	**1.05 (1.05-1.06)**	HR per 5 units	***p* < 0.000**	**1.04 (1.04-1.05)**
			HR per 10 units	***p* < 0.000**	**1.11 (1.10-1.12)**	HR per 10 units	***p* < 0.000**	**1.08 (1.08-1.09)**

All hazard ratios (HRs) and its 95% confident intervals (CIs) were derived from Cox proportional hazards regression. The analyses were adjusted for age, sex ethnicity, BMI, educational level, Townsend Deprivation Index and sedentary behaviour.

Table S5 The association between different smoking-related exposures and incident back pain after excluding participants with self-reported chronic back pain

Smoking Status			Number of cigarettes smoked daily			Pack-Years of own Smoking		
Categories	n/events	HR (95%CI)	Categories	n/events	HR (95%CI)	Categories	n/events	HR (95%CI)
Never	209734/13657	1 (ref)	None	256776/16855	1 (ref)	None	258756/17051	1 (ref)
Former	133838/10251	**1.13 (1.10-1.16)**	Up to 10	32672/2462	**1.12 (1.08-1.17)**	Up to 10	30790/2061	1.04 (0.99-1.08)
Current	27176/2709	**1.52 (1.46-1.59)**	Between 11 and 20	59425/5114	**1.24 (1.20-1.28)**	Between 11 and 20	30475/2407	**1.16 (1.11-1.21)**
P for trend	***p* < 0.000**	**1.19 (1.17-1.22)**	Between 21 and 30	14540/1411	**1.39 (1.31-1.46)**	Between 21 and 30	21726/2002	**1.33 (1.25-1.37)**
			More than 30	7335/775	**1.44 (1.34-1.55)**	More than 30	29001/3096	**1.46 (1.40-1.52)**
			P for trend	***p* < 0.000**	**1.11 (1.10-1.12)**	P for trend	***p* < 0.000**	**1.10 (1.09-1.11)**
			HR per 5 units	***p* < 0.000**	**1.05 (1.05-1.06)**	HR per 5 units	***p* < 0.000**	**1.04 (1.04-1.04)**
			HR per 10 units	***p* < 0.000**	**1.11 (1.10-1.12)**	HR per 10 units	***p* < 0.000**	**1.08 (1.07-1.09)**

All hazard ratios (HRs) and its 95% confident intervals (CIs) were derived from Cox proportional hazards regression. The analyses were adjusted for age, sex ethnicity, BMI, educational level, Townsend Deprivation Index and sedentary behaviour.
